# Influence of the lateral pterygoid muscle on traumatic temporomandibular joint bony ankylosis

**DOI:** 10.1186/s12903-016-0220-1

**Published:** 2016-05-28

**Authors:** Tian-ge Deng, Chang-kui Liu, Ping Liu, Lin-lin Zhang, Li-geng Wu, Hong-zhi Zhou, Yu-xiang Ding, Kai-jin Hu

**Affiliations:** State Key Laboratory of Military Stomatology, National Clinical Research Center for Oral Diseases, Shaanxi Clinical Research Center for Oral Diseases, Department of Oral Surgery, School of Stomatology, The Fourth Military Medical University, 145 West Changle Road, Xi’an, 710032 China; Department of Stomatology, 451th Hospital of the People’s Liberation Army, 269 East Youyi Road, Xi’an, 710049 China; Department of Endodontics and Restorative Dentistry, School of Stomatology, Tianjin Medical University, Tianjin, 300070 China

**Keywords:** Animal model, Distraction osteogenesis, Lateral pterygoid muscle, Sagittal fracture of mandibular condyle, Temporomandibular joint ankylosis

## Abstract

**Background:**

The pathogenesis of traumatic TMJ ankylosis remains unclear. This study aimed to verify the role of the lateral pterygoid muscle in the pathogenesis of traumatic temporomandibular joint (TMJ) bony ankylosis.

**Methods:**

Eight 6-month-old male sheep were used in this study. Bilateral TMJ osteotomies were performed to induce sagittal fractures of the mandibular condyle. The lateral one-fourth segment of the disc was removed to establish a model of TMJ bony ankylosis. Subsequently, the function of the left and right lateral pterygoid muscles was blocked (experimental group) or maintained (control group), respectively. At 12 weeks postoperatively, animals were sacrificed and TMJ complex samples were evaluated by gross observation, spiral computed tomography (CT), micro-CT, and histological examinations.

**Results:**

Gross observation revealed bony ankylosis in the control TMJs and fibrous adhesions in the experimental TMJs. Spiral CT and micro-CT demonstrated that, compared to the experimental group, the control group showed calcified callus formation in the joint space and roughened articular surfaces after new bone formation, which protruded into the joint space. Maximum mediolateral and anteroposterior condylar diameters were significantly larger in the control group than in the experimental group. Micro-CT also showed that the primary growth orientation of new trabeculae was consistent with the direction of lateral pterygoid traction in the control group, but not in the experimental group. Histological examination showed fibro-osseous ankylosis in the control group, but not in the experimental group.

**Conclusions:**

The lateral pterygoid simulates the effects of distraction osteogenesis, which is an important factor in the pathogenesis of TMJ bony ankylosis during the healing of sagittal condylar fractures.

## Background

Temporomandibular joint (TMJ) ankylosis is characterized by stiffening of the joint due to abnormal adhesion and rigidity of the relevant bones after injury or disease. This condition has significant effects on physical and mental health and results in a poor quality of life, with symptoms including trismus, masticatory difficulty, speech impairment, and pain [1–5]. In clinical practice, the most common cause of TMJ ankylosis is trauma, with sagittal fracture of the mandibular condyle (SFMC) being the primary etiology [2, 4, 5]. The treatment of TMJ ankylosis is difficult, although various treatment techniques have been reported [1, 2, 4–7]. It has been hypothesized that traumatic TMJ ankylosis occurs after condylar fracture [4, 5, 7], although the organization and ossification of an intracapsular hematoma secondary to TMJ injury has also been thought to cause this condition [8, 9]. Thus, the pathogenesis of traumatic TMJ ankylosis remains unclear.

In 1982, Rowe found that TMJ ankylosis can result from SFMC [10], while Duan et al. reported that type III sagittal fractures were associated with disc displacement, indicating a high risk of TMJ ankylosis [11]. He et al. reported that 25 of 40 cases of TMJ ankylosis were caused by sagittal fractures [7]. Moreover, animal studies have revealed that sagittal fractures could result in severe secondary TMJ injury and, consequently, progressive changes toward TMJ ankylosis [12]. Recently, many experimental and clinical studies have suggested that disc rupture/displacement and traumatic TMJ ankylosis are closely related [4, 9]. Yan et al. suggested that severe damage to the glenoid fossa plays an important role in the development of traumatic TMJ bony ankylosis [13]. However, the relationship between traction of the lateral pterygoid muscle and traumatic TMJ ankylosis is not well understood.

Distraction osteogenesis was first described by Ilizarov in the 1950s, who suggested that bony regeneration occurs in the gap between the fractured segments through gradual distraction, based on osteotomy or corticotomy and an appropriate distraction force [4]. The inferior head of the lateral pterygoid muscle inserts into the pterygoid fovea, below the condylar process of the mandible. In the case of SFMC, the traction from the lateral pterygoid muscle pulls the fractured segment anteriorly and medially. We have previously demonstrated that, during the fracture healing process, this traction results in overgrowth of new bone between the fragment and the lateral stump of the condyle; however, it does not result in TMJ bony ankylosis [9]. Taken together, the findings of the abovementioned studies suggest that traumatic TMJ bony ankylosis is the outcome of several associated factors. Therefore, we hypothesized that TMJ ankylosis is caused by overgrowth of new bone secondary to the distraction osteogenesis effect of the lateral pterygoid muscle during the process of SFMC healing, in addition to damage to the articular disc and glenoid fossa. The present study aimed to establish an animal model in which SFMC is combined with damage to the articular disc and glenoid fossa, to verify whether disjunction of the lateral pterygoid muscle prevents traumatic TMJ bony ankylosis. Such information would aid the development of appropriate treatment approaches for SFMC that can prevent traumatic TMJ ankylosis.

## Methods

### Ethics committee approval and animals

The experimental protocol was approved by the Ethics Committee of the State Key Laboratory of Military Stomatology, National Clinical Research Center for Oral Diseases, Shaanxi Clinical Research Center for Oral Diseases, School of Stomatology, Fourth Military Medical University, Xi’an, China (SYXK 2015–001).

Eight 6-month-old (weight: 20–30 kg), healthy male sheep were used in the present study. The animals were cared for according to the guidelines set by the Laboratory Animal Research Centre of Fourth Military Medical University. They were caged for a week prior to surgery to ensure the absence of abnormalities in occlusion or in their TMJs. All right-sided TMJs were considered control specimens, while the left joints were used as test specimens.

### Surgical procedures

**Fig. 1:**
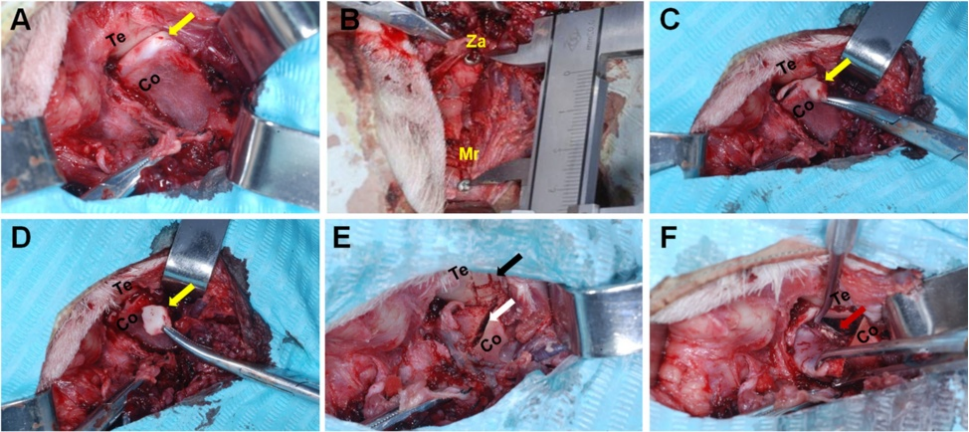
Surgical procedures in the control (**a**–**e**) and experimental groups (**a**–**f**). **a** Exposure of the temporomandibular joint (TMJ) complex. Te: temporal bone, Co: condylar process, yellow arrow: articular disc. **b** Measurement of the preoperative ramus height in both groups. Mr: mandibular ramus, Za: zygomatic arch. **c** Cutting of the anterior and posterior disc attachments. Te: temporal bone, Co: condylar process, yellow arrow: articular disc. **d** Removal of the lateral one-fourth of the articular disc. Te: temporal bone, Co: condylar process, yellow arrow: lateral one-fourth of the articular disc. **e** Placement of grid grooves measuring 2 columns × 2 rows using an ultrasound osteotome on the surface of the glenoid fossa. White arrow: the sagittal fracture line of the mandibular condyle from the lateral pole of the condyle to the medial side of the condylar neck. Te: temporal bone, Co: condylar process. Black arrow: grid grooves measuring “2 × 2” in the shape of a “#”, representing “2 columns × 2 rows”. **f** Removal of the medial lateral pterygoid muscle attachment on the fracture segments (red arrow: broken ends of the lateral pterygoid muscle attachment) (Te: temporal bone, Co: condylar process)

General anesthesia was induced by intramuscular injection of xylazine hydrochloride (0.1 mL/kg; Founder Animal Pharmaceutics, Ji Lin, China). The vertical maximum mouth opening was measured and recorded. In the control group, the right temporal and preauricular regions were shaved and disinfected. The region was isolated with sterile drapes, and lidocaine was injected as a local anesthetic. A 6-cm-long curved preauricular incision was made, the skin flap was raised, and the TMJ complex was exposed (Fig. 1a). To measure changes in the surgical height of the mandibular ramus on both sides, the zygomatic arch and mandibular ramus were exposed at the periphery of the TMJ complex. One titanium screw was fixed on each of the zygomatic arch and the mandibular ramus, approximately 2.0–3.0 cm away from the TMJ, and the distance between the two screws was measured and recorded (Fig. 1b). The capsule was exposed by blunt dissection, and the inferior joint space was exposed by horizontal incision through the capsule at the condylar neck. The condylar head was isolated using a periosteal elevator, and the superior joint space was exposed by separation of the lateral attachment of the disc through the inferior joint space. The anterior and posterior attachments of the disc were then cut off (Fig. 1c) and the lateral one-fourth of the articular disc was removed (Fig. 1d). An oblique vertical osteotomy was performed from the lateral pole of the control condyle to the medial side of the condylar neck, using an ultrasound osteotome, to simulate an SFMC (type B intracapsular fracture). Grid grooves measuring 2 columns × 2 rows (approximately 1 mm deep) were carved, using an ultrasound osteotome, on the surface of the glenoid fossa (Fig. 1e). In addition, the lateral pterygoid muscles were maintained on the internal poles of the fractured condyles in the control group. The capsule was not sutured, and the wound was closed in layers [9].

In the experimental group, approximately 0.5–1.0 cm of the lateral pterygoid muscle was removed surgically (Fig. 1f). The other surgical procedures were the same as for the control group. After surgery, each sheep was administered an antibiotic (penicillin, 20 mg/kg; X-Y Biotechnology, Shanghai, China) and an analgesic (pentazocine, 1 mg/kg) for 5 days.

### Sample preparation

After surgery and before sacrifice, wound healing and the eating behavior of the sheep were monitored. One day before sacrifice, the maximum mouth opening was measured and recorded under general anesthesia, after which spiral computed tomography (CT) was performed (120 kV, 80 mA, 0.8-s rotation time, 0.2-mm slice thickness, GE Medical Systems, Milwaukee, WI, USA) using a GE Light-speed 32-slice CT scanner. Multiplanar reconstruction was used to generate CT images of the TMJ complexes. Morphological features, including the shape and erosion pattern of the temporal and condylar surfaces, and calcification in the joint space were evaluated.

At 12 weeks after surgery, all sheep were sacrificed by intravenous injection of a lethal dose of pentobarbital sodium (>0.5 g/kg). The TMJ complexes were dissected and the distance between the two positioned titanium screws were again determined. The TMJ complexes were immediately removed en bloc using a slow speed diamond saw (Northwest Medical Equipment, Shan Xi, China), cleaned by stripping the flesh, and fixed in 10 % buffered formalin for 72–96 h.

### Micro-CT

Micro-CT images of the TMJ complexes were obtained using a preclinical cone-beam CT scanner (Healthcare Explore Locus SP, GE Medical Systems, Milwaukee, WI, USA), which is a CCD-based imaging tool that acquires data by capturing a number of planar images at a regular angular velocity. The images were acquired using the following parameters: X-ray tube voltage, 80 kV; anode current, 80 μA; exposure time, 3000 ms; binning combination, 1 × 1; rotation angle, 360°; angle increment, 0.4°; and scanning revolution rate, 21 μm. The trabecular microarchitecture was evaluated using the built-in software of the micro-CT device, with direct three-dimensional (3D) morphometry for 3D image reconstruction. The center of the fractured area was selected as the region of interest (ROI; 3 × 3 × 3 mm).

A model-independent method was used to quantify various architectural parameters, including the bone volume fraction (BV/TV), trabecular number (Tb.N), trabecular thickness (Tb.Th), trabecular separation (Tb.Sp), and the degree of anisotropy (DA). BS/BV was obtained using a plate model, and DA was derived using the mean intercept length method. DA correlates with the orientation of the loading regime of the bone, and the trabecular bone in the load-bearing skeletal regions adapts to transmit compressive or tensile loads along its primary orientation [14–16]. These parameters are typically used to evaluate bone quality. The ROI was set at the center of the fractured area and was automatically calculated using Micview V2.1.2 and Advanced Bone Analysis software (Healthcare Explore Locus SP, GE Medical Systems, Milwaukee, WI, USA).

### Histological examinations

After micro-CT scanning, all TMJ complex specimens were divided into three blocks by coronal sectioning: anterior, central, and posterior. The central block was embedded in 5 % benzoyl peroxide (Eastern Chemical Reagent, Chong Qing, China) and polymerized. The hardened tissue was sliced into 25-μm-thick sections using a hard tissue microtome (2500E, LEICA, Wetzlar, Germany) and subjected to Van Gieson staining.

### Statistical analysis

SPSS 17.0 software (SPSS Inc., Chicago, IL, USA) was used for statistical analysis. Paired-sample *t*-tests were used to compare the maximum mouth opening, change in the mandibular ramus height, and microarchitectural parameters between the control and experimental TMJ groups. 8 parameters (8 *p*-values) were designed for the Tables 1, 2, 3 in the present study, a significance test was performed for each one, so all in all 8 test. Multiple significance testing was corrected by Bonferroni, a *P*-value of < 0.00625 (0.05/8) was considered statistically significant.

**Table 1:** Maximal mouth opening (MMO) of the eight sheep before and after surgical induction of sagittal fracture of the mandibular condyle (mean±SD)

	Before surgery	After surgery	*P*
MMO [cm]	4.78 ± 0.42	2.33 ± 0.28	<0.001

**Table 2:** Distance between the titanium screws on the zygomatic arch and mandibular ramus, indicating the ramus height, during and after surgery for induction of sagittal fracture of the mandibular condyle, in the control and experimental groups (mean ± SD)

	Control group		Experimental group		*P*
	During surgery	After surgery	During surgery	After surgery	
^a^Distance [cm]	2.99 ± 0.52	2.47 ± 0.43	3.14 ± 0.23	3.13 ±0.23	0.046

^a^distance between titanium screws positioned on the zygomatic arch and mandibular ramus

Control group: lateral pterygoid muscle function maintained on the right side

Experimental group: lateral pterygoid muscle function blocked on the side

**Table 3:** Microarchitectural parameters in the fractured condylar segment in the control and experimental groups (mean ± SD)

Parameters	12 weeks		
	Control group	Experimental group	*P*
DA [%]	1.61 ± 0.05	1.16 ± 0.02	<0.001
BV/TV [%]	0.41 ± 0.04	0.49 ± 0.05	0.001
Tb. Th [mm]	0.59 ± 0.10	0.47 ± 0.05	0.005
Tb.N [1/mm]	3.23 ± 0.27	2.59 ± 0.10	<0.001

DA: degree of anisotropy, BV/TV: bone volume fraction, Tb Th: trabecular thickness, Tb.N: trabecular, BS/BV: bone surface to volume ratio, Tb.Sp: trabecular separation

Control group: lateral pterygoid muscle function maintain on the rifgt side

## Results

### Gross observation findings

All sheep tolerated surgery and exhibited spontaneous recovery. No wound infection was observed, and the cut lateral pterygoid muscle in the experimental group did not reconnect. At 12 weeks after surgery, the mean maximum mouth opening was significantly smaller than that before surgery (*P* < 0.00625; Table 1). There was no statistical significance in the mandibular ramus height in the control and experimental groups (*P* > 0.00625; Table 2).

**Fig. 2:**
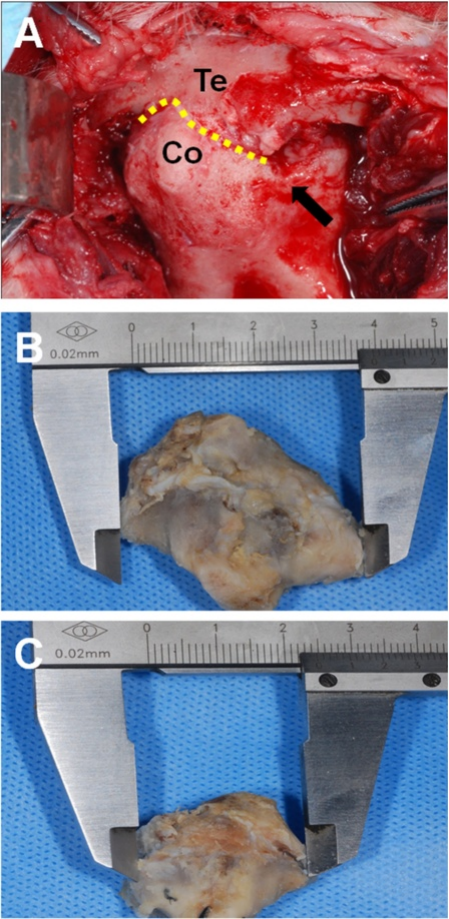
Gross observation of the temporomandibular joint (TMJ) complexes at 12 weeks after surgery. **a** TMJ bony ankylosis in the control group. Te: temporal bone, Co: condylar process. Dotted yellow line: bony fusion in the TMJ bony ankylosis. Black arrow: Y-shaped bifid condyle. **b** Maximum mediolateral diameter of the control condyle. **c** Maximum mediolateral diameter of the experimental condyle

In the control group, TMJ bony ankylosis at the lateral articular surface and Y-shaped bifid condyles could be observed by the naked eye during dissection (Fig. 2a). On isolation of the TMJ complexes, we observed new bone formation, with an irregular pattern, on the articular surfaces in both groups. The maximum mediolateral and anteroposterior condylar diameters were larger in the control group than in the experimental group (Figs. 2b, c). In the medial region with an existing disc, the articular cartilage and glenoid fossa did not exhibit fusion.

In the experimental group, fibrous adhesions were observed in the joint space, although TMJ bony ankylosis was absent.

### Spiral CT findings

**Fig. 3:**
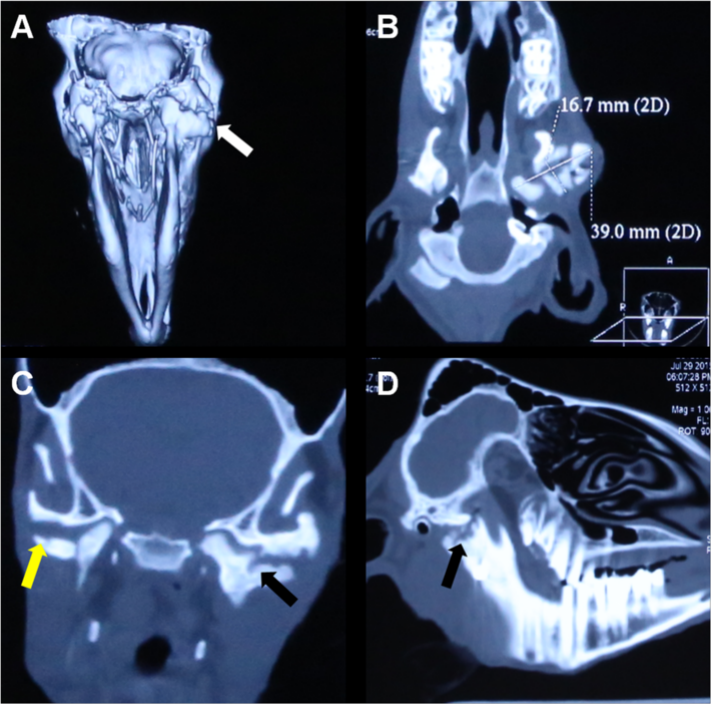
Spiral computed tomography (CT) analysis of the temporomandibular joint (TMJ) complexes at 12 weeks after surgery. **a** Three-dimensional reconstruction of a spiral CT image shows more newly formed bone in the control group (white arrow) than in the experimental group. **b** A horizontal CT section shows that the maximum mediolateral and anteroposterior condylar diameters are larger in the control group than in the experimental group. **c** and **d** Coronal and sagittal CT sections show irregular upper and lower articular surfaces, calcified bone callus formation in the joint space (black arrow), a narrowed joint space, and roughened articular surfaces after new bone formation, which protrudes into the joint space in the control group (black arrow). The experimental group shows irregular erosion on the upper and lower articular surfaces (yellow arrow), and the joint space is clear with no intra-articular calcification

Spiral CT revealed more new bone formation in the control group than in the experimental group (Fig. 3a). The maximum mediolateral and anterioposterior diameters were larger in the control group than in the experimental group at 12 weeks after surgery (Fig. 3b).

In the control group, the joints showed irregular upper and lower articular surfaces, Y-shaped bifid condyles, calcified callus formation in the joint space, a narrowed joint space (with nearly complete disappearance of the lateral joint space). Articular surfaces were roughened after the formation of new bone, which protruded into the joint space (Fig. 3c, d). TMJ bony ankylosis occurred at 12 weeks after surgery.

In the experimental group, the joint space was almost normal and clear, with no intra-articular calcification, although irregular erosion was observed on the upper and lower articular surfaces. No TMJ bony ankylosis had occurred at 12 weeks after surgery.

### Micro-CT findings

The microarchitectural data are shown in Table 3. All parameters were significantly different between the two groups at 12 weeks after surgery (*P* < 0.00625).

**Fig. 4:**
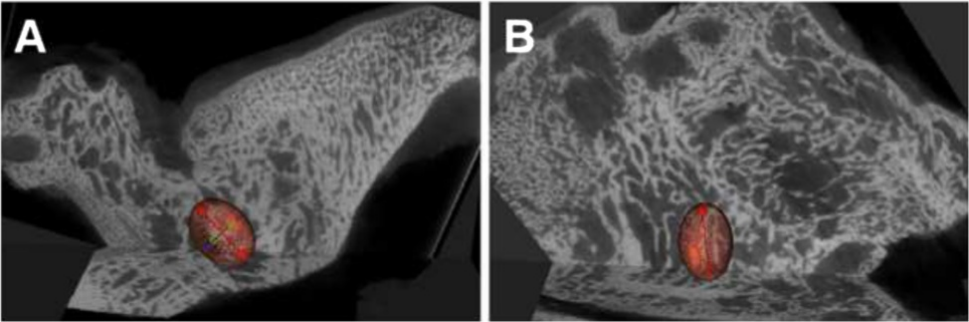
Micro-computed tomography (CT) analysis of the temporomandibular joint (TMJ) complexes at 12 weeks after surgery (coronal section analysis). **a** Evaluation of the direction of the trabeculae in the control group. The trabecular direction (red Y-axis) is consistent with the direction of traction of the lateral pterygoid muscle. **b** Evaluation of the trabecular direction in the experimental group. The trabecular direction (red Y-axis) is inconsistent with the direction of traction of the lateral pterygoid muscle

DA, which represents the main direction of new trabecular bone in the fractured segment, was consistent with the direction of traction of the lateral pterygoid muscle in the control group (Fig. 4a), but not in the experimental group (Fig. 4b).

Moreover, as compared with that in the experimental group, the lateral joint space was filled with new cartilage and bone at the lateral articular surface in the control group. TMJ bony ankylosis and Y-shaped bifid condyles were observed, consistent with spiral CT findings.

### Histological examination findings

**Fig. 5:**
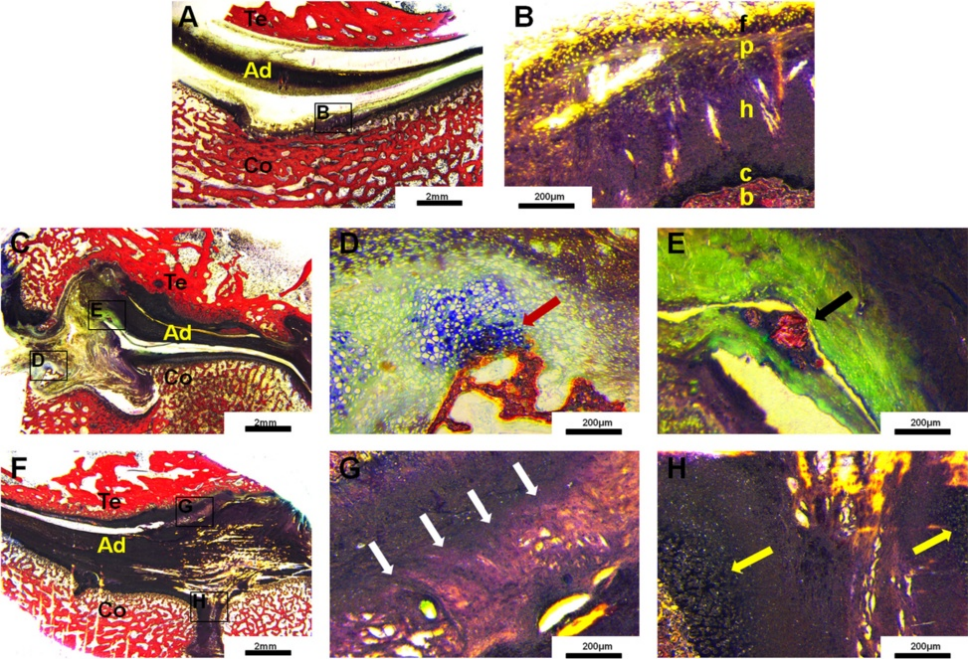
**a** and **b** Histological sections of the normal temporomandibular TMJ complexes (coronal section analysis). **a** The normal histological features of the TMJ complexes are illustrated (Van Gieson staining). Te: temporal bone, Ad: articular disc, Co: condylar process. **b** The fibrocartilage layer on the top surface of the condyle can be differentiated into five layers: fibrous, proliferative, hypertrophic, calcified, and trabecular layers, marked as **f,** **p,** **h,** **c,** and **b** (Van Gieson staining). **c**–**e** Histological section of the control TMJ complexes at 12 weeks afyer surgery (coronal section analysis). **c** Fibro-osseous ankylosis in the control group (Van Gieson staining). Te: temporal bone, Ad: articular disc, Co: condylar process. **d** The joint space is composed of abundant neoformative cartilaginous matrix, cartilage cells (red arrow) and a small amount of fibrous tissue (Van Gieson staining). **e** Neoformative endochondral ossification (black arrow) can be seen (Van Gieson staining). **f**–**h** Histological section of the experimental TMJ complexes at 12 weeks after surgery (coronal section analysis). **f** The joint space is filled with abundant fibrous tissue (Van Gieson staining). Te: temporal bone, Ad: articular disc, Co: condylar process. **g** There are very few cartilaginous cells and little matrix in the joint space. The joint space is filled with considerable fibrous tissue, with partial fibrous adhesion between the glenoid fossa and articular disc (white arrow; Van Gieson staining). **h** Regeneration and remodeling are shown in the fractured segment. The fibrous layer adjacent to the fracture site is thicker, and new cartilage cell clusters are seen in the hypertrophic layer (yellow arrow; Van Gieson staining)

The histological features of the normal TMJ complex are illustrated in Fig. 5a. The surface of the glenoid fossa was composed of dense fibrous tissue in the coronal sections, and the top surface of the normal condyle could be differentiated into five layers (Fig. 5b).

In the control group, fibro-osseous ankylosis was observed, and the joint space was filled with abundant new cartilaginous matrix, cartilage cells, and a small amount of fibrous tissue, with endochondral ossification present at 12 weeks after surgery (Fig. 5c–e).

In the experimental group, the joint space was filled with a considerable amount of fibrous tissue, but no neoformative cartilage cells and matrix were present. Regeneration and remodeling were observed in the fractured region. The fibrous layer adjacent to the fracture site appeared thicker, and new cartilage cell clusters were observed in the hypertrophic layer at 12 weeks after surgery (Fig. 5f–h).

## Discussion

Traumatic TMJ ankylosis has been associated with several risk factors, including some types of condylar fractures, disc rupture/displacement, severe TMJ damage, and prolonged immobilization of the mandible [7, 17, 18]. Animal experiments and clinical observations have shown that SFMC or type B intracapsular fractures are more serious risk factors for TMJ ankylosis than are other types of condylar fractures [4, 7, 17, 19, 20]. Therefore, we here established an animal model in which SFMC was combined with articular disc and glenoid fossa damage. We found that blockade of the function of the lateral pterygoid muscle prevented the development of traumatic TMJ bony ankylosis, while maintaining the function of this muscle successfully established an animal model of traumatic TMJ bony ankylosis.

Yan et al. [17] reported that, in all animal models, development of traumatic TMJ bony ankylosis required several conditions, including condylar damage, articular disc rupture/displacement, and glenoid fossa damage or bone grafting in the joint space. Our previous experiment verified the presence of considerable overgrowth of new bone in the group in whom the lateral pterygoid muscle function was maintained, but we found that no TMJ bony ankylosis occurred [9]. Therefore, we modified our previous protocol in the present study to include the simulation of SFMC in the control TMJ (in which the right lateral pterygoid muscle function was maintained) and experimental TMJ groups (in which the function of the left lateral pterygoid muscle was blocked), as well as minor damage to the glenoid fossa, and removal of the lateral one-fourth of the disc to induce TMJ bony ankylosis. We observed that only the TMJ complexes in which the lateral pterygoid muscle function was maintained developed TMJ bony ankylosis.

Thus, our findings demonstrated that traumatic TMJ bony ankylosis was associated with several factors. Because TMJ bony ankylosis was not observed in the absence of damage to the glenoid fossa in our previous study, we believe that glenoid fossa damage is an important factor in the development of TMJ bony ankylosis, consistent with the findings of Yan et al. [13]. However, in the present study, despite similar glenoid fossa damage on both sides, only the TMJ complexes in which lateral pterygoid muscle function was maintained developed bony ankylosis, while those in which the lateral pterygoid muscle function was blocked did not. These findings suggest that the lateral pterygoid muscle is an important factor in the development of traumatic TMJ bony ankylosis.

Passive maximum mouth opening has been accepted as an important indicator of TMJ ankylosis in animal models [13]. In the present study, the maximum mouth opening of all animals had decreased significantly by 12 weeks after surgery. Radiographic and histological examinations also demonstrated new cartilage and bone formation in the joint space in the control group. All these findings suggested the successful induction of TMJ bony ankylosis in this model.

We analyzed the trabecular bone microarchitecture in the fractured segment. The DA parameter in micro-CT analysis is essential for the detection of alterations in the trabecular structure [21, 22]. According to Wolff’s law, trabecular cancellous bone is arranged in the direction of the dominant stress, and when the loading stress changes, the trabecular bone is rearranged according to the new stress trajectory. Anisotropic trabeculae result from application of load in one or a few orientations, while isotropic trabeculae result from application of load in several directions [22–24]. Increased trabecular anisotropy in the fractured segment reflects preferential preservation of the trabeculae that are oriented in the direction of the dominant stress, while decreased trabecular anisotropy reflects recessive preservation of the trabeculae that are oriented in the direction of the dominant stress [16, 24–26].

In the present study, DA was significantly higher in the control group than in the experimental group. The longest axis of the ellipsoid of DA was consistent with the direction of the traction vector of the lateral pterygoid muscle in the control group, which underlies the parallel direction of the primary trabeculae and the direction of the traction vector of the lateral pterygoid muscle. This has also been observed in other studies on trabecular bone in the superolateral femoral neck [25, 26]. In addition, histological examinations in our previous study showed that new trabeculae in the fractured segment were regularly arranged in a particular direction, i.e., the direction of the traction vector of the lateral pterygoid muscle, in the group in which this muscle function was retained [27]. Furthermore, gross observation and radiographic and histological examinations revealed greater overgrowth of new bone in the control group of the present study. These findings verified that new bone overgrowth is caused by the distraction osteogenesis effect of the lateral pterygoid muscle during the process of SFMC healing, in addition to disc and glenoid fossa damage, which together eventually led to traumatic TMJ bony ankylosis.

In the present study, we observed typical Y-shaped bifid condyles in the area of the TMJ complexes, which are associated with traumatic TMJ ankylosis [5, 28, 29]. Clinical observations and other animal experiments have also reported similar results in the past [5, 29]. We also attributed this Y-shaped bifid condyle formation to the distraction osteogenesis effect of the lateral pterygoid muscle during the SFMC healing process. When SFMC occurs, the medial head and lateral ramus stump of the fractured condyle separate. Then, the medial fractured head is displaced anteromedially by traction of the lateral pterygoid muscle, which results in the formation of a typical Y-shaped bifid condyle [28].

Consistent with other clinical observations and animal experiments, we observed that TMJ bony ankylosis usually occurred at the lateral articular surface [5, 6, 13, 17]. In the present study, we simulated B-type intracapsular fractures, and we found that the vertical height of the mandibular ramus decreased after surgery, as indicated by the change in the distance between the two titanium screws, although there was no statistical significance in the mandibular ramus height between the control and experimental groups, maybe the power of test was not enough. Furthermore, clinical observations showed the decrease in the stump height of the mandibular ramus in most patients of TMJ bony ankylosis [7]. We deduced that the stump height of the mandible ramus decreased during the SFMC healing process, because displacement of the condyle allowed the lateral surface of the ramus stump to approach the temporal surface more closely, mediated by the function of the jaw elevator muscle, and thus increasing the likelihood of transarticular bony fusion, which was similar to that in other studies [4, 7, 8, 30]. In addition, medial dislocation of the fractured condylar head through traction from the lateral pterygoid muscle created a cavity that was rapidly filled with blood, creating a large hematoma that subsequently ossified [8, 9]. This is an important factor for bony bridge formation in TMJ ankylosis at the lateral articular surface.

The present study showed that the distraction osteogenesis effect of the lateral pterygoid muscle may be an important factor in the pathogenesis of traumatic TMJ bony ankylosis during the healing of SFMCs. These findings will further guide the clinical treatment of mandibular condylar fractures (e.g., by wearing open-mouth plates/occlusal pads) and will help to prevent TMJ ankylosis. Yang et al. [9] used an open-mouth plate to treat four children with SFMC and found an almost normal radiological morphology after treatment. Our previous study [2] also showed satisfactory outcomes of SFMC treatment by means of occlusal pads, which caused relaxation of the lateral pterygoid muscle and blockade of its function, thus decreasing the incidence of TMJ ankylosis.

## Conclusions

In conclusion, the results of the present study suggest that the lateral pterygoid muscle simulates the effects of distraction osteogenesis during the process of SFMC healing, and that this distraction osteogenesis effect is an important factor in the pathogenesis of traumatic TMJ bony ankylosis during this healing process.

## Abbreviations

BS/BV, bone surface to volume ratio; BV/TV, bone volume fraction; CT, computed tomography; DA, degree of anisotropy; SFMC, sagittal fracture of the mandibular condyle; Tb.N, trabecular number; Tb.Th, trabecular thickness; Tb.Sp, trabecular separation; TMJ, temporomandibular joint

## References

[1] Hag J, Patel N, Weimer K, Matthews NS (2014). Single stage treatment of the temporomandibular joint using patient-specific total joint replacement and virtual surgical planning. Br J Oral Maxillofac Surg.

[2] Liu CK, Meng FW, Tan XY, Xu J, Liu HW, Liu SX (2014). Clinical and radiological outcomes after treatment of sagittal fracture of mandibular condyle (SFMC) by using occlusal splint in children. Br J Oral Maxillofac Surg.

[3] Wang YL, Li XJ, Qin RF, Lei DL, Liu YP, Wu GY (2008). Matrix metalloproteinase and its inhibitor in temporomandibular joint osteoarthrosis after indirect trauma in young goats. Br J Oral Maxillofac Surg.

[4] Meng FW, Zhao JL, Hu KJ, Liu YP (2009). A new hypothesis of mechanisms of traumatic ankylosis of temporomandibular joint. Med Hypotheses.

[5] Long X, Gross AN (2007). Pathological change after the surgical creation of a vertical intracapsular condylar fracture. Int J Oral Maxillofac Surg.

[6] Li JM, An JG, Wang X, Yan YB, Xiao E, He Y (2014). Imaging and histologic features of traumatic temporomandibular joint ankylosis. Oral Surg Oral Med Oral Pathol Oral Radiol.

[7] He D, Ellis E, Zhang Y (2008). Etiology of temporomandibular joint ankylosis secondary to condylar fractures: the role of concomitant mandibular fractures. J Oral Maxillofac Surg.

[8] Ferretti C, Bryant R, Becker P, Lawrence C (2005). Temporomandibular joint morphology following post-traumatic ankylosis in 26 patients. Int J Oral Maxillofac Surg.

[9] Liu CK, Liu P, Meng FW, Deng BL, Xue Y, Mao TQ (2012). The role of the lateral pterygoid muscle in the sagittal fracture of mandibular condyle (SFMC) healing process. Br J Oral Maxillofac Surg.

[10] Rowe NL (1982). Ankylosis of the temporomandibular joint. J R Coll Surg Edinb.

[11] Duan DH, Zhang Y (2011). A clinical investigation on disc displacement in sagittal fracture of the mandibular condyle and its association with TMJ ankylosis development. Int J Oral Maxillofac Surg.

[12] Long X, Goss AN (2007). A sheep model of intracapsular condylar fracture. J Oral Maxillofac Surg.

[13] Yan YB, Zhang Y, Gan YH, An JG, Li JM, Xiao E (2013). Surgical induction of TMJ bony ankylosis in growing sheep and the role of injury severity of the glenoid fossa on the development of bony ankylosis. J Craniomaxillofac Surg.

[14] Okazaki N, Chiba K, Taquchi K, Nango N, Kubota S, Ito M (2014). Trabecular microfractures in the femoral head with osteoporosis: analysis of microcallus formations by synchrotron radiation micro CT. Bone.

[15] Willems NM, Mulder L, Langenbach GE, Grünheid T, Zentner A, van Eijden TM (2007). Age-related changes in microarchitecture and mineralization of cancellous bone in the porcine mandibular condyle. J Struct Biol.

[16] Monje A, Monje F, Gonzalez-Garcia R, Galindo-Moreno P, Rodriquez-Salvanes F, Wang HL (2014). Comparison between microcomputed tomography and cone-beam computed tomography radiologic bone to assess atrophic posterior maxilla density and microarchitecture. Clin Oral Implants Res.

[17] Yan YB, Liang SX, Shen J, Zhang JC, Zhang Y (2014). Current concepts in the pathogenesis of traumatic temporomandibular joint ankylosis. Head Face Med.

[18] Laskin DM (1978). Role of the meniscus in the etiology of posttraumatic temporomandibular joint ankylosis. Int J Oral Surg.

[19] Meng F, Hu K, Kong L, Zhao Y, Liu Y, Zhou S (2010). Veterinary and radiological evaluations of open and closed treatment of type-B diacapitular (intracapsular) fractures of the mandibular condyle in sheep. Br J Oral Maxillofac Surg.

[20] Meng FW, Hu KJ, Kong L, Zhao YT, Liu YP, Zhou SX (2009). Morphological evaluation of temporomandibular joint after open and closed treatment of type B diacapsular condylar fractures in sheep. Ann Anat.

[21] Siu WS, Qin L, Cheung WH, Leung KS (2004). A study of trabecular bones in ovariectomized goats with micro-computed tomography and peripheral quantitative computed tomography. Bone.

[22] Majumdar S, Genant HK, Grampp S, Newitt DC, Truong VH, Lin JC (1997). Correlation of trabecular bone structure with age, bone mineral density, and osteoporotic status: in vivo studies in the distal radius using high resolution magnetic resonance imaging. J Bone Miner Res.

[23] Fajardo RJ, Müller R (2001). Three-dimensional analysis of nonhuman primate trabecular architecture using micro-computed tomography. Am J Phys Anthropol.

[24] Ford CM, Keaveny TM (1996). The dependence of shear failure properties of trabecular bone on apparent density and trabecular orientation. J Biomech.

[25] Sinclair KD, Farnsworth RW, Pham TX, Knight AN, Bloebaum RD, Skedros JG (2013). The artiodactyl calcaneus as a potential ‘control bone’ cautions against simple interpretations of trabecular bone adaptation in the anthropoid femoral neck. J Hum Evol.

[26] Milovanovic P, Djonic D, Marshall RP, Hahn M, Nikolic S, Zivkovic V (2012). Micro-structural basis for particular vulnerability of the superolateral neck trabecular bone in the postmenopausal women with hip fractures. Bone.

[27] Wu D, Yang XJ, Cheng P, Deng TG, Jiang X, Liu P (2015). The lateral pterygoid muscle affects reconstruction of the condyle in the sagittal fracture healing process: a histological study. Int J Oral Maxillofac Surg.

[28] Li Z, Djae KA, Li ZB (2011). Post-traumatic bifid condyle: the pathogenesis analysis. Dent Traumatol.

[29] Rehman TA, Gibikote S, Ilango N, Thaj J, Sarawagi R, Gupta A (2009). Bifid mandibular condyle with associated temporomandibular joint ankylosis: a computed tomography study of the patterns and morphological variations. Dentmaxillofac Radiol.

[30] He D, Cai Y, Yang C (2014). Analysis of temporomandibular joint ankylosis caused by condylar fracture in adults. J Oral Maxillofac Surg.

